# Cancer diagnosis marker extraction for soft tissue sarcomas based on gene expression profiling data by using projective adaptive resonance theory (PART) filtering method

**DOI:** 10.1186/1471-2105-7-399

**Published:** 2006-09-04

**Authors:** Hiro Takahashi, Takeshi Nemoto, Teruhiko Yoshida, Hiroyuki Honda, Tadashi Hasegawa

**Affiliations:** 1 Department of Biotechnology, School of Engineering, Nagoya University, Furo-cho, Chikusa-ku, Nagoya 464-8603, Japan; 2 Research Fellow of the Japanese Society for the Promotion of Science (JSPS), Japan; 3 Genetics Division, National Cancer Center Research Institute, 5-1-1 Tsukiji, Chuo-ku, Tokyo 104-0045, Japan; 4 Pathology Division, National Cancer Center Research Institute, 5-1-1 Tsukiji, Chuo-ku, Tokyo 104-0045, Japan; 5 Department of Surgical Pathology, Sapporo Medical University School of Medicine, South 1 West 16, Chuo-ku, Sapporo 060-8543, Japan

## Abstract

**Background:**

Recent advances in genome technologies have provided an excellent opportunity to determine the complete biological characteristics of neoplastic tissues, resulting in improved diagnosis and selection of treatment. To accomplish this objective, it is important to establish a sophisticated algorithm that can deal with large quantities of data such as gene expression profiles obtained by DNA microarray analysis.

**Results:**

Previously, we developed the projective adaptive resonance theory (PART) filtering method as a gene filtering method. This is one of the clustering methods that can select specific genes for each subtype. In this study, we applied the PART filtering method to analyze microarray data that were obtained from soft tissue sarcoma (STS) patients for the extraction of subtype-specific genes. The performance of the filtering method was evaluated by comparison with other widely used methods, such as signal-to-noise, significance analysis of microarrays, and nearest shrunken centroids. In addition, various combinations of filtering and modeling methods were used to extract essential subtype-specific genes. The combination of the PART filtering method and boosting – the PART-BFCS method – showed the highest accuracy. Seven genes among the 15 genes that are frequently selected by this method – *MIF*, *CYFIP2*, *HSPCB*, *TIMP3*, *LDHA*, *ABR*, and *RGS3 *– are known prognostic marker genes for other tumors. These genes are candidate marker genes for the diagnosis of STS. Correlation analysis was performed to extract marker genes that were not selected by PART-BFCS. Sixteen genes among those extracted are also known prognostic marker genes for other tumors, and they could be candidate marker genes for the diagnosis of STS.

**Conclusion:**

The procedure that consisted of two steps, such as the PART-BFCS and the correlation analysis, was proposed. The results suggest that novel diagnostic and therapeutic targets for STS can be extracted by a procedure that includes the PART filtering method.

## Background

Soft tissue sarcomas are a group of highly heterogeneous tumors that exhibit a diverse spectrum of mesenchymal differentiations. However, the molecular dissection of tumor heterogeneity has been hampered by the relatively low incidence of these tumors; approximately 3,800 cases are reported annually in Japan. Significant differences were observed in the five-year survival rates among the subtypes of STS, *e.g*., 100% for well-differentiated liposarcoma (WLS), 71% for synovial sarcoma (SS), 46% for pleomorphic malignant fibrous histiocytoma (MFH), and 92% for myxofibrosarcoma (MFS). The primary objective of this study was to identify a set of marker genes that facilitates accurate differential diagnosis of the sarcoma subtypes. Discrimination between MFH and MFS, for example, is particularly difficult because there is a histological overlap between the two. Information on such subtype-specific genes may also help in understanding the molecular pathways that are activated in each subtype of the different biological malignancies.

Recent advances in DNA microarray analysis have enabled the simultaneous evaluation of the expression levels of several tens of thousands of genes, thereby offering a rich source of information that is potentially useful in the diagnosis and prognosis of diseases [1]. There are two main methods of expression data analyses: unsupervised learning methods and supervised learning methods. The unsupervised learning methods, *e.g*., hierarchical clustering [2] and fuzzy adaptive resonance theory (Fuzzy ART) [3], are designed to identify previously unrecognized classes of disease based on their expression pattern; the biological significance of such disease subtypes, such as prognosis, is then assessed. In contrast, the supervised learning methods use training sets to specify the genes that should be clustered together [4]. However, to conduct either unsupervised or supervised analysis, it is necessary to select genes that have a strong correlation with the target phenotype, such as disease diagnosis or prognosis. This is because the performance of classification analysis can decline if a large number of genes as predictor variables are incorporated in the model.

Gene selection has been performed to screen candidate genes for modeling. There are two types of approaches – the wrapper approach and the filtering approach. In the former approach, genes are selected as a part of mining algorithms, such as k-nearest neighbor (kNN), multiple regression analysis (MRA), weighted voting (WV) [5], support vector machines (SVM) [6], fuzzy neural network (FNN) combined with SWEEP operator (FNN-SWEEP) method [7], and boosted fuzzy classifier with SWEEP operator (BFCS) method [8,9]. On the other hand, in the latter approach, prior to the application of the mining algorithms, genes are selected by filtering methods, such as the Mann-Whitney U test, Student's t-test (Sttest), Welch's t-test (Wttest), the signal-to-noise statistic (S2N) [5], significance analysis of microarrays (SAM) [10], nearest shrunken centroids (NSC) [11], and the projective adaptive resonance theory (PART) filtering method [12].

In a previous study, we developed the PART filtering method by modifying PART [13,14], and reported that PART exhibited a higher performance than conventional methods, such as S2N and NSC [12]. The combination method of PART and BFCS (PART-BFCS) was developed and applied to gene expression data, such as lymphoma [15] and esophageal cancer [16]. In the present study, we applied the various filtering methods to the gene expression profile data for the STS subtypes and constructed SVM models using the filtered genes. The results showed that the accuracy of the model based on the genes filtered by PART was the highest. In addition, various wrapper methods were applied to the genes that were filtered by the different filtering methods to extract essential genes for diagnosis. The models of the PART-BFCS method among various combinations of filtering and wrapper methods showed the highest accuracy, and 28 independent probes were extracted using this method. Seven genes among the 15 probes that were frequently selected by this method are known prognostic marker genes for other tumors. These genes are candidate marker genes for STS. Correlation analysis was performed for the 15 genes to extract the subtype-specific genes that were not selected by PART-BFCS. Sixteen genes among those extracted are also known prognostic marker genes for other tumors, and these could be candidate marker genes for STS.

## Results and discussion

### Clustering analysis for unfiltered data

**Figure 1:**
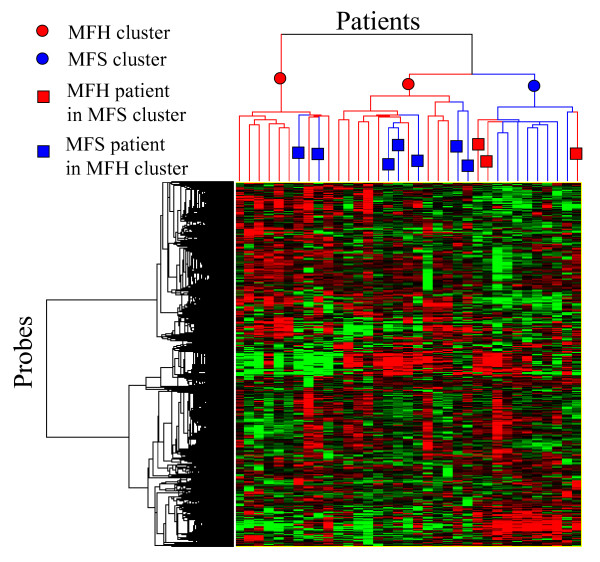
Hierarchical clustering of STS patients by using 12,241 unfiltered probes.

Hierarchical clustering was applied to 35 patients and 12,241 unfiltered probes, as shown in Fig. 1. Figure 1 shows that patients were separated into three clusters – two MFH clusters and a single MFS cluster. However, there were seven MFS patients in the MFH clusters and three MFH patients in the MFS cluster that were misclassified by the clustering. On the basis of these results, various filtering and wrapper methods were performed for a more accurate separation of these patients.

### Construction of SVM models by using filtered genes

To eliminate nonspecific genes for discriminating between MFH and MFS, various filtering methods, such as the U test, Sttest, Wttest, S2N, SAM, NSC, and PART were applied to the modeling data set comprising 26 patients and 12,241 probes; the performances were evaluated by using prediction accuracies for the blind data. The top 1,000 genes selected by each filtering method were used to construct the SVM models. The blind accuracies of models for each method are shown in Table 1. Table 1 shows that the accuracy of the SVM model using genes filtered by PART, which was 88.8%, was the best in this study. The accuracies of models using S2N or SAM (77.7%) were the second highest. On the other hand, the accuracy of the SVM model without filtering was 55.6%, which was the lowest. Average accuracy of the models with random selection was also 55.6%. These results suggest that when constructing diagnostic models, it is necessary to incorporate a filtering step; further, in this study, the PART filtering method was found to give the most accurate predictions.

### Application of various combinations of filtering and wrapper methods

To extract essential subtype-specific genes for differentiation between MFH and MFS, various wrapper methods such as kNN, MRA, WV, SVM, FNN-SWEEP, and BFCS were applied to the modeling data set comprising 26 patients and 1,000 probes filtered by each filtering method; the performances were evaluated by using the prediction accuracies of the blind data. The genes selected by each wrapper method were used in the models, and numbers of inputs were optimized by cross-validation of the modeling data set. The blind accuracies were calculated by using ten combination models that were constructed by PIM, as shown in Table 2. Table 2 shows that the average accuracy of PART-BFCS was 81.1%, which was the highest. There was a total of 80 probes in ten combinations of 8-input models. Some probes were selected several times. Among 80 probes, 28 were independent. The average accuracies of the SAM-kNN and PART-SVM methods, at 74.4% and 73.3%, were the second and third best, respectively. These results imply that the combination of PART and BFCS is the most accurate method for extraction of essential subtype-specific genes for STS.

### Clustering analysis using genes extracted by PART-BFCS

**Figure 2:**
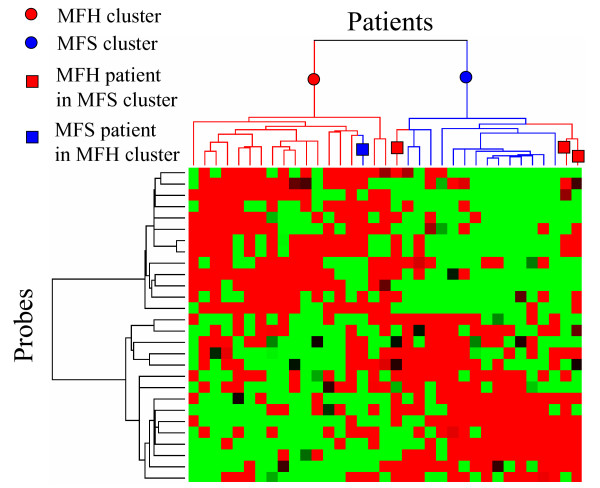
Hierarchical clustering of STS patients by using 28 genes selected by PART-BFCS.

Hierarchical clustering was applied to 35 patients and 28 probes selected by PART-BFCS, as shown in Fig. 2. Figure 2 shows that patients were separated into two clusters – an MFH cluster and an MFS cluster. The results show that there was a single MFS patient in the MFH cluster and three MFH patients in the MFS cluster. These observations suggest that misclassification of samples was reduced using the genes that were extracted by the PART-BFCS method and that essential genes could be extracted for the diagnosis of STS subtypes.

### Extraction of marker gene candidates by the correlation analysis

**Figure 3:**
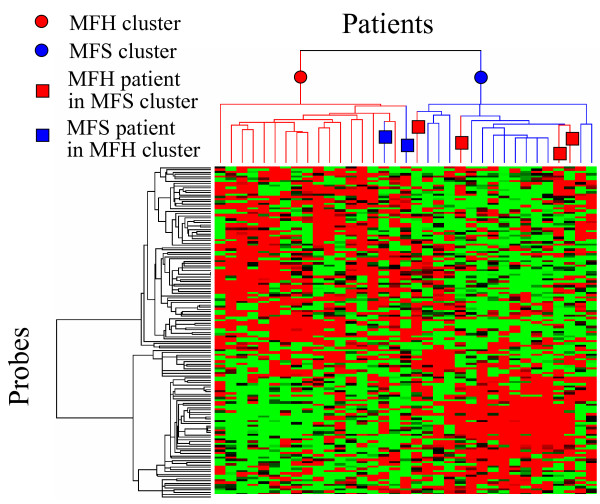
Hierarchical clustering of STS patients by using 145 probes having high correlation with the 15 probes selected by PART-BFCS.

To extract the marker gene candidates unextracted by PART-BFCS, the correlation analysis was applied to STS data. Twenty-eight probes were extracted by PART-BFCS. Fifteen probes among 28 ones were selected two times or more. As shown in Table 3, a total of 150 probes, comprising the top 10 probes having high correlation with the 15 probes, were extracted as marker gene candidates. Some probes were selected several times. Thus, these probes comprised 145 independent probes, which correspond to 126 independent genes. The performance of the 145 probes was confirmed by hierarchical clustering, as shown in Fig. 3. Figure 3 shows that patients were separated into two clusters – an MFH cluster and an MFS cluster. The results show that there was two MFS patient in the MFH cluster and four MFH patients in the MFS cluster. This result was almost the same as Figure 2. This is, the genes that have high performances, were extracted by using correlation analysis.

### Characteristics of the genes selected for the classification models and the genes highly correlated with them

Significant differences were observed in the five-year survival rates between MFH and MFS. Thus, it was expected that prognostic marker genes would be extracted for the discrimination of MFH and MFS. We investigated the presence of previously reported prognostic marker genes among the 15 probes (genes) selected frequently by PART-BFCS among the 28 probes. Furthermore, 145 probes which correspond to 126 independent genes, were investigated.

With regard to the genes selected directly by PART-BFCS, seven genes among the 15 genes are reported to be prognostic markers for other tumors. *MIF *promotes tumor invasion and metastasis via the Rho dependent pathway, reported by Sun *et al*. [17]. *CYFIP2 *(*PIR121*) is one of the genes downregulated by p53, reported by Ceballos *et al*. [18]. p53 is a well-known type of tumor suppressor gene. *HSPCB *plays an important role in the assembly/disassembly of tubulin by inhibiting tubulin polymerization, reported by Man *et al*. [19]. Tubulin is a simple and useful predictive marker for the clinical response to chemotherapy in gastric cancer, reported by Urano *et al*. [20]. Reduced expression of *TIMP3 *in esophageal adenocarcinoma is associated with increased tumour invasiveness and reduced patient survival, reported by Darnton *et al*. [21]. *LDHA *is a hypoxia-inducible gene and is associated with considerably poorer overall survival, reported by Chi *et al*. [22]. *ABR *is a regulator of the Rho GTP-binding protein family, reported by Chuang *et al*. [23]. The Rho pathway is associated with tumor invasion and metastasis, reported by Sun *et al*. [17]. *RGS3 *is associated with tumor metastasis, reported by Tatenhorst *et al*. [24]. These findings suggest that the genes extracted by the PART-BFCS method are new marker genes for the STS subtypes.

With regard to the genes selected by correlation analysis, sixteen genes among 126 genes are reported to be prognostic markers for other tumors. The ADD3 protein (adducinγ) belongs to a family of ubiquitously expressed membrane-skeletal proteins that are localized at spectrin-actin junctions, reported by van den Boom *et al*. [25]. In renal carcinomas, changes in adducin expression, phosphorylation state, and localization were found to be associated with increased malignancy. In addition, the down-regulation of adducin-γ expression is correlated with increased migratory activity of human glioma cells in vitro. The expression of *COL11A1 *in colorectal tumors could be associated with the APC/β-catenin pathway in familial adenomatous polyposis (FAP) and sporadic colorectal cancer, reported by Fischer *et al*. [26]. Nuclear accumulation of the beta-catenin protein is associated with activation of the Wnt/Wg signaling pathway. Beta-catenin status predicts a favorable outcome in childhood medulloblastoma, reported by Ellison *et al*. [27]. *SMAD3 *is a component of the transforming growth factor-beta (TGFβ), which is a potent regulator of growth, apoptosis, and invasiveness of tumor cells, such as breast cancer cells, reported by Dubrovska *et al*. [28]. TGFβ1/SMAD3 suppresses BRCA1-dependent DNA repair in response to DNA damaging agents. *GAS7*, a growth arrest-specific gene, is the partner gene of *MLL *in treatment-related acute myeloid leukemia. *MLL *gene translocations can be present early during anticancer treatment at low cumulative doses of DNA topoisomerase II inhibitors, reported by Megonigal *et al*.[29]. *CD130 *(*IL6ST*) expression is associated with disease activity in multiple myeloma, reported by Barille *et al*. [30]. *MMP1 *expression is correlated significantly with the evolution of lymph node status and tumor-lymph node-metastasis (TNM) stage, reported by Gouyer *et al*. [31]. Expression of *MMP9 *and *MMP13 *is positively associated with poor tumor cell differentiation, vessel permeation, and lymph node metastasis, reported by Gu *et al*. [32]. *MMP11 *(*ST3*) is associated with lymph node involvement and tumor progression, reported by Soni *et al*. [33]. *TSSC3 *is one of the genes related to apoptosis, reported by Muller *et al*. [34]. *HSPB2 *(*HSP27*) is implicated in resistance to chemotherapy in breast cancer, and also predicts a poor response to chemotherapy in leukemia patients, reported by Ciocca and Calderwood [35]. *HSP105B *is an alternatively spliced form of *HSP105A*, reported by Yamagishi *et al*. [36]. *HSP105A *prevents stress-induced apoptosis in neuronal PC12 cells, and it is a novel anti-apoptotic neuroprotective factor in the mammalian brain. An anti-ICAM2 monoclonal antibody induces immune-mediated regressions of ICAM2-negative colon carcinomas, reported by Melero *et al*. [37]. *HSPD1 *is downregulated during early apoptosis of hepatoma cells, reported by Lee *et al*. [38]. *WNT10B *is a member of the WNT signaling molecules, which are potent targets for the diagnosis of cancer (susceptibility, metastasis, and prognosis) as well as for the prevention and treatment of cancer, reported by Kirikoshi and Katoh[39]. *TEK *is correlated with a higher risk of metastases in node-negative patients, reported by Dales *et al*. [40]. Thus, correlation analysis was performed to extract the subtype-specific genes that were not selected by PART-BFCS. These findings suggest that the genes having a high correlation with those extracted by the PART-BFCS method could also be new marker genes for the STS subtypes, and that this fact gives greater confidence in the accuracy of these potential maker genes selected directly by PART-BFCS.

## Conclusion

In this study, we applied the PART filtering method to STS gene expression profiling data to construct subtype predictors for diagnosis. The results showed that the genes selected by PART exhibited higher prediction accuracy for STS than the other methods assessed. The genes selected by PART-BFCS such as *MIF*, *CYFIP2*, *HSPCB*, *TIMP3*, *LDHA*, *ABR*, and *RGS3 *can be used as targets for molecular diagnosis and treatment. In addition, the new candidate marker genes that were not extracted directly by PART-BFCS, could be extracted by correlation analysis. We believe that this procedure, the PART filtering method, should be considered as one of the candidate analytical procedures in various class prediction problems in clinical and basic oncology using transcriptome data.

## Methods

### Microarray analysis

The gene expression profile data were obtained from 35 surgical specimens of STS – 20 pleomorphic malignant fibrous histiocytomas (MFH) and 15 myxofibrosarcomas (MFS). For RNA extraction, trained pathologists carefully excised the tissue samples from the main tumor, leaving a margin clear from the surrounding non-tumorous tissue. Microscopically, the samples may still contain several non-tumor cells such as infiltrating lymphocytes, tissue macrophages, and vascular and lymphatic endothelial cells. However, unlike carcinomas, it is difficult to eliminate non-tumor stroma in case of soft tissue sarcomas; therefore, laser microdissection was not performed in this study. Total RNAs extracted from the bulk tissue samples were biotin-labeled and hybridized to high-density oligonucleotide microarrays (Affymetrix Human Genome U133A 2.0 Array) comprising 22,283 probe sets representing 18,400 transcripts, according to the manufacturer's instructions. The scanned array data were processed by Affymetrix Microarray Suite v.5.1, which scaled the average intensity of all the genes on each array to the target signal of 1,000.

### Data processing

In this experiment, the data set was randomly partitioned into two groups – 26 samples (15 MFH and 11 MFS) as a modeling data set for constructing the subtype prediction model (predictor) and nine samples (5 MFH and 4 MFS) as a blind data set for evaluating the constructed predictor. Validations were performed by comparing the accuracies in the blind data set, instead of cross-validation accuracies, as reported by Bhasin and Raghava [41]. In the present study, cross-validation was used to optimize various parameters of the models for the modeling data.

In the 35 specimens, the probes that expressed at a signal intensity of less than 1,000 were excluded as a preprocess procedure prior to the application of various combinations of filtering and modeling methods. It is empirically difficult to reproduce the expression by RT-PCR for the genes which have signal intensity of less than 1,000, when their gene expression values were scaled to target signal of 1,000. Accordingly, 12,241 probes were selected. During the gene filtering step, 1,000 probes were selected using each filtering method. For each filtering method, SVM models were constructed to differentiate between MFH and MFS by using the filtered genes. In addition, various wrapper methods were used to extract essential genes for diagnosis; these are described in the following sections.

With regard to the wrapper methods, the parameter increasing method (PIM) [42] was used to select input combinations for model construction in the modeling methods. To validate the performance of the models, 10 independent combination models were constructed. The accuracy of the subtype prediction of the blind data was also calculated as the average of 10 combination predictors.

### Model construction with parameter selection

The PIM was used to select input combinations for the construction of kNN, MRA, WV, SVM, and FNN-SWEEP models. This was conducted as follows:

Firstly, we predicted the subtype of each sample by using a prediction model with a single input. Prediction models for each probe were constructed in a series, and all the probes were ordered based on the accuracy of the constructed models. In the next step, the probe having the highest accuracy was used for constructing a combination model.

Secondly, we selected a partner probe for the probe selected in the first step in order to increase the prediction accuracy. To accomplish this, we constructed a 2-input model in which a ranked probe was designated as input 1, and input 2 (the partner probe) was selected to provide the highest training accuracy; doing so, we applied FNN-SWEEP (kNN, MRA, WV, SVM, or SVM) and PIM to the modeling data. By repeating this step, a combination of *N*_opt _(optimized by leave-one-out cross-validation of the modeling data) candidate probes was identified for use as input probes in the model construction.

Finally, an *N*_opt _input model was constructed. The probes with the 1st to the 10th highest accuracies were used as the first inputs for the construction of the 10 combination models by PIM. The performance of the prediction models was evaluated by applying them to the blind data set.

### Fuzzy neural network (FNN) combined with the SWEEP operator method (FNN-SWEEP)

The FNN-SWEEP method was also applied for model construction. The FNN-SWEEP method was originally proposed by Noguchi *et al*. [43] and modified by Ando *et al*. [7] to manage microarray data. The FNN has three types of weight parameters (*w*_*c*_, *w*_*g*_, and *w*_*f*_) [44]. For the FNN-SWEEP method, only parameter *w*_*f *_was optimized by the SWEEP operator method at the gene selection step. After the input combinations were determined, FNN models with selected input combinations were optimized using a backpropagation algorithm at the model construction step. For backpropagation, the number of epochs was set to 5,000 and the learning rate was set to 0.1; these values are the same as those reported by Ando *et al*. [7].

### Support vector machine (SVM)

The SVM was originally proposed by Vapnik and Chervonenkis [45] and is used to prevent the "curse of dimensionality." The SVM is superior to many conventional methods and is frequently used in bioinformatics. In the present study, the SVM-LIGHT software package [46] was used. This software was modified, and the PIM function was added to select for a combination of inputs. The regulatory parameter *c *was the default value of SVM_LIGHT ((avg. (input vector)^2^)^-1^). A linear kernel was used because a similar cross-validation accuracy of the model was obtained for the modeling data set using various kernels.

### Boosted fuzzy classifier with SWEEP operator (BFCS)

BFCS is a type of advanced AdaBoost algorithm [47]. The BFCS algorithm has been described previously [8]. Briefly, multiple single-input predictors were first constructed by the FNN-SWEEP method. Then, BFCS was used to calculate adequate weights for the weak predictors, and the weighted weak predictors were assembled efficiently. As a result, the integrated predictor could correctly classify as many samples as possible by minimizing and smoothing out the probability of making an error in each individual sample.

### k-nearest neighbor (kNN)

kNN methods are based on a *distance function*, such as the Euclidean distance, for pairs of tumor samples. The kNN proceeds as follows to classify blind data set observations on the basis of the modeling data set. For each patient in the blind data set, (a) it finds the *k *closest patients in the modeling data set and (b) it predicts the class by majority vote; that is, it chooses the class that is most common among those *k *neighbors. The number of neighbors *k *was chosen as three because a similar cross-validation accuracy of the model was obtained in the modeling data set for various values of *k*.

### Multiple regression analysis (MRA)

MRA is a conventional method of statistical analysis. The MRA can be used to describe and evaluate the relationship between the subtypes of tumor and gene expression. MRA models were used to help us predict the subtypes of cancer by using gene expression data.

### Weighted voting (WV)

The WV method was originally proposed by Golub *et al*. [5] to manage microarray data. The weights of each gene were calculated by the signal-to-noise ratio. The linear models of one gene were assembled with gene weight.

### Hierarchical clustering analysis

Hierarchical clustering is widely used as one of the unsupervised learning methods. This clustering method was applied to the STS subtype analysis by using CLUSTER software [2] for the cases of the 12,241 unfiltered probes or the 28 probes selected by PART-BFCS. In this study, hierarchical clustering was performed by using centroid-linkage.

### Correlation analysis

Correlation analysis was performed to extract the subtype-specific genes of the STS that were not selected by PART-BFCS. Correlation coefficients for the 15 genes that were selected two times or more by PART-BFCS were calculated by Pearson's correlation coefficient.

## Authors' contributions

HT developed the software, analyzed microarray data, and wrote the manuscript. NE carried out experiment of microarray. TY, HH, and TH conceived of the study, and participated in its design and coordination. All authors read and approved the final manuscript.

## Figures and Tables

**Table 1:** Blind accuracies for the SVM models using different filtering methods

Filtering method	Number of genes	Accuracy (%) SVM model
PART	1000	88.9
NSC	1000	66.7
S2N	1000	77.8
SAM	1000	77.8
Student's t-test	1000	66.7
U-test	1000	66.7
Welch's t-test	1000	66.7
Random selection^1^	1000	55.6
No filtering	12241	55.6

^1 ^The SVM model was constructed by using 1000 probes selected randomly. This process was repeated 1000 times. Average accuracies of 1000 SVM models were calculated.

**Table 2:** Blind accuracies for various combinations of filtering and modeling methods

Filtering methods	Wrapper methods					
	
	BFCS	FNN	SVM	MRA	kNN	WV
PART	81.1 ± 14.1 (8)	64.4 ± 15.6 (3)	73.3 ± 5.4 (2)	60.0 ± 16.6 (11)	67.8 ± 16.8 (10)	56.7 ± 13.6(14)
NSC	68.9 ± 6.7 (5)	60.0 ± 12.4 (3)	62.2 ± 13.3 (3)	65.6 ± 9.2 (9)	68.9 ± 13.0 (3)	66.7 ± 9.9 (21)
S2N	68.9 ± 6.7 (15)	56.7 ± 16.1 (3)	61.1 ± 15.9 (3)	61.1 ± 14.3 (4)	63.3 ± 17.2 (4)	58.9 ± 12.2 (18)
SAM	71.1 ± 7.4 (12)	64.4 ± 12.0 (3)	67.8 ± 13.6 (3)	63.3 ± 12.2 (10)	74.4 ± 8.7 (7)	63.3 ± 11.2 (9)
Student's t-test	71.1 ± 5.4 (15)	53.3 ± 12.0 (4)	60.0 ± 10.2 (13)	58.9 ± 13.2 (5)	68.9 ± 8.3 (4)	60.0 ± 19.4 (26)
U-test	66.7 ± 9.9 (9)	56.7 ± 16.1 (3)	64.4 ± 13.0 (7)	54.4 ± 10.2 (7)	67.8 ± 11.6 (14)	62.2 ± 12.4 (1)
Welch's t-test	65.6 ± 10.5 (15)	55.6 ± 14.9 (3)	58.9 ± 8.7 (13)	53.3 ± 12.2	67.8 ± 10.5	65.6 ± 13.6 (12)
No filtering	68.9 ± 9.7 (10)	58.9 ± 10.0 (3)	66.7 ± 15.7 (2)	61.1 ± 13.4 (3)	55.6 ± 15.7 (3)	57.8 ± 17.1 (26)

Parenthesized values indicate the numbers of probes used in each model.

**Table 3:** The genes selected by PART-BFCS and the genes having high correlation with them

Accession no.	Gene name	Times of selection	Top 10 high correlation genes									
			
			1	2	3	4	5	6	7	8	9	10
NM_002415	MIF	9	NIPSNAP1 NM_003634 (0.74)	DDT NM_001355 (0.73)	PORIMIN BG538627 (0.73)	NDUFA7 NM_005001 (0.71)	SNAP29 NM_004782 (0.71)	TSSC3 AF001294 (0.71)	MMP1 NM_002421 (0.71)	LSM1 NM_014462 (0.71)	DGCR14 L77566 (0.71)	MRPL20 NM_017971 (0.69)
AB032261	SCD	8	SCD AA678241 (0.94)	SMAP1 NM_021940 (0.74)	SCD BC005807 (0.69)	NRBF-2 AA883074 (0.67)	INSIG1 NM_005542 (0.65)	FADS1 AL512760 (0.63)	VDAC2 L08666 (0.63)	GLUD1 NM_005271 (0.62)	TFG NM_006070 (0.62)	TFAP2A BF343007 (0.61)
NM_016332	SEPX1	7	SIAH2 U76248 (0.81)	WHSC1 BF111870 (0.73)	KIAA0220 AI424872 (0.73)	BAIAP3 AI799802 (0.71)	TBC1D1 AB029031 (0.70)	FARSLA AD000092 (0.70)	OPRS1 NM_005866 (0.70)	CTBP2 N23018 (0.70)	TCF3 AW062341 (0.69)	KCTD5 NM_018992 (0.68)
AL161999	CYFIP2	5	CRISPLD2 AL136861 (0.91)	NTF3 NM_002527 (0.90)	PRO1331 NM_030778 (0.89)	TNFSF11 AF053712 (0.88)	NTRK3 S76476 (0.87)	SLC24A3 NM_020689 (0.86)	KCNIP1 NM_014592 (0.86)	CP NM_000096 (0.86)	ARTN AF120274 (0.86)	KIAA0523 AB011095 (0.86)
AI218219	HSPCB	5	HSPCB AF275719 (0.89)	HSP105B NM_006644 (0.84)	HSP105B BG403660 (0.82)	FOXG1B NM_005249 (0.81)	HSPD1 BE256479 (0.80)	TERA_ NM_021238 (0.80)	DNAJB1 BG537255 (0.79)	HSPE1 NM_002157 (0.79)	FXR1 AI990766 (0.79)	NXT2 AF201942 (0.78)
AI811298	OSR2	5	OAZ AW149417 (0.80)	FXYD1 NM_005031 (0.78)	FBLN2 NM_001998 (0.78)	PMP22 L03203 (0.73)	KIAA0763 AI652645 (0.73)	TEKNM_000459 (0.73)	KIAA0644 NM_014817 (0.72)	GAS7 BE439987 (0.71)	FLJ10159 NM_018013 (0.70)	WNT10B NM_003394 (0.69)
U67195	TIMP3	5	TIMP3 BF347089 (0.91)	TIMP3 AW338933 (0.90)	IL6ST AW242916 (0.88)	IL6ST NM_002184 (0.88)	IL6ST AB015706 (0.82)	HLA-DRB3 AA807056 (0.80)	TIMP3 NM_000362 (0.78)	IL6ST BE856546 (0.76)	HAS1 NM_001523 (0.74)	C6orf133 AB002347 (0.74)
NM_020122	PCMF	4	NTPBP AB044661 (0.85)	MGC10882 BC004952 (0.80)	C16orf34 AK023154 (0.77)	FKBP4 NM_002014 (0.77)	PFDN2 NM_012394 (0.76)	FKBP4 AA894574 (0.75)	LDLR NM_000527 (0.75)	STIP1 BE886580 (0.74)	AHSA1 NM_012111 (0.74)	FXR1 NM_005087 (0.73)
NM_001998	FBLN2	4	GAS7 NM_005890 (0.86)	GAS7 BE439987 (0.86)	PMP22 L03203 (0.85)	BMP1 NM_006129 (0.79)	OSR2 AI811298 (0.78)	KIAA0644 NM_014817 (0.77)	FXYD1 NM_005031 (0.77)	WNT10B NM_003394 (0.74)	ZDHHC3 NM_016598 (0.73)	AHNAK BG287862 (0.73)
NM_005566	LDHA	4	PLOD2 NM_000935 (0.74)	ALDOA AK026577 (0.72)	ADM NM_001124 (0.70)	PSMA1 NM_002786 (0.69)	ALDOA NM_000034 (0.68)	VDAC1 AL515918 (0.68)	QSCN6 NM_002826 (0.67)	PKM2 NM_002654 (0.67)	PSG3 BC005924 (0.67)	TCP11L1 NM_018393 (0.67)
NM_005756	GPR64	3	ADD3 NM_019903 (0.81)	ADD3 AI818488 (0.79)	SLC4A4 NM_003759 (0.79)	ADD3 AI763123 (0.79)	ADD3 BE545756 (0.78)	CRYAB AF007162 (0.78)	LRRC16 NM_017640 (0.77)	EYA2 U71207 (0.74)	HSPB2 NM_001541 (0.71)	SPRY1 BF508662 (0.71)
AL136663	PLXNA1	2	PCBP2 AW103422 (0.70)	MGC5566 NM_024049 (0.63)	CLIC5 NM_016929 (0.63)	SMAD3 NM_015400 (0.62)	PTPRB NM_002837 (0.62)	SMAD3 BF971416 (0.62)	ICAM2 AA126728 (0.62)	SEMA3G NM_020163 (0.61)	KIAA0417 AB007877 (0.61)	EXT1 NM_000127 (0.60)
AL136663	ABR	2	RNMTL1 NM_018146 (0.72)	MFAP4 R72286 (0.62)	ABR AL136663 (0.61)	KIAA1085 AU160676 (0.61)	P2RX4 NM_002560 (0.61)	CYP2E AF182276 (0.60)	LOC51031 AF061730 (0.60)	ZNF212 NM_012256 (0.59)	GSPT2 NM_018094 (0.59)	IDUA NM_000203 (0.59)
AL527773	RARRES2	2	PANX1 NM_015368 (0.91)	CCT8 NM_006585 (0.89)	GART NM_000819 (0.88)	ASMTL Y15521 (0.87)	ASMTL AA669799 (0.87)	ASMTL BC002508 (0.87)	SERPINB7 NM_003784 (0.85)	SERPINB3 AB046400 (0.84)	SERPINB4 U19557 (0.83)	ATP5O NM_001697 (0.83)
NM_021106	RGS3	2	TDO2 NM_005651 (0.84)	MMP13 NM_002427 (0.81)	COL11A1 NM_001854 (0.80)	MMP9 NM_004994 (0.75)	COL11A1 J04177 (0.74)	CLECSF5 NM_013252 (0.72)	HBA2 T50399 (0.72)	SLC19A1 AF004354 (0.72)	MMP11 AI761713 (0.71)	MMP11 NM_005940 (0.71)
13 additional genes												

The left hand side of the table shows the genes selected by PART-BFCS and the right hand side shows the genes correlated with them. Parenthesized values indicate correlation coefficients.
